# Model-driven user interfaces for bioinformatics data resources: regenerating the wheel as an alternative to reinventing it

**DOI:** 10.1186/1471-2105-7-532

**Published:** 2006-12-14

**Authors:** Kevin Garwood, Christopher Garwood, Cornelia Hedeler, Tony Griffiths, Neil Swainston, Stephen G Oliver, Norman W Paton

**Affiliations:** 1 School of Computer Science, University of Manchester, Oxford Road, Manchester M13 9PL, UK; 2 Manchester Centre for Integrated Systems Biology, Manchester Interdisciplinary Biocentre, University of Manchester,131 Princess Street, Manchester M1 7DN, UK; 3 Faculty of Life Sciences, University of Manchester, Michael Smith Building, Oxford Road, Manchester M13 9PT, UK

## Abstract

**Background:**

The proliferation of data repositories in bioinformatics has resulted in the development of numerous interfaces that allow scientists to browse, search and analyse the data that they contain. Interfaces typically support repository access by means of web pages, but other means are also used, such as desktop applications and command line tools. Interfaces often duplicate functionality amongst each other, and this implies that associated development activities are repeated in different laboratories. Interfaces developed by public laboratories are often created with limited developer resources. In such environments, reducing the time spent on creating user interfaces allows for a better deployment of resources for specialised tasks, such as data integration or analysis. Laboratories maintaining data resources are challenged to reconcile requirements for software that is reliable, functional and flexible with limitations on software development resources.

**Results:**

This paper proposes a model-driven approach for the partial generation of user interfaces for searching and browsing bioinformatics data repositories. Inspired by the Model Driven Architecture (MDA) of the Object Management Group (OMG), we have developed a system that generates interfaces designed for use with bioinformatics resources. This approach helps laboratory domain experts decrease the amount of time they have to spend dealing with the repetitive aspects of user interface development. As a result, the amount of time they can spend on gathering requirements and helping develop specialised features increases. The resulting system is known as Pierre, and has been validated through its application to use cases in the life sciences, including the PEDRoDB proteomics database and the e-Fungi data warehouse.

**Conclusion:**

MDAs focus on generating software from models that describe aspects of service capabilities, and can be applied to support rapid development of repository interfaces in bioinformatics. The Pierre MDA is capable of supporting common database access requirements with a variety of auto-generated interfaces and across a variety of repositories. With Pierre, four kinds of interfaces are generated: web, stand-alone application, text-menu, and command line. The kinds of repositories with which Pierre interfaces have been used are relational, XML and object databases.

## Background

The discipline of bioinformatics makes use of varied data resources that provide access to both experimental data and the results of analyses over such data. There are many hundred resources in the public domain [1]; individual laboratories maintain their own collections by either building on local data or collecting relevant results of specific studies.

Duplication of facilities occurs amongst interfaces to many such repositories. Typical functionalities include the ability to:

• browse top-level collections in a repository, such as listing experiments recorded in a microarray database;

• evaluate simple parameterised searches over the contents of repositories, such as using thresholds when running identification algorithms over mass spectrometric data;

• use predicates applied to selected fields to construct searches over repository collections, such as using resolution values to retrieve only certain structures from a protein database;

• and evaluate queries over repositories using query languages specific to those repositories.

Repositories do not often provide all capabilities, and some repositories provide specialised interfaces, such as data-specific graphical viewers. However, where capabilities are similar amongst interfaces, these interfaces are routinely constructed in a bespoke manner.

The central role of data repositories in bioinformatics seems secure, given the ever-growing numbers of high-throughput experimental techniques. Also, there will be a need for laboratories to manage large and increasing quantities of locally produced experimental data before any associated analyses can be submitted for publication. Furthermore, the bioinformatics community increasingly encourages its members to produce data sets that may be more easily exchanged within the community than has previously been the case. To further this aim, various organisations are developing standards using models that describe the storage, structure and content of laboratory data. The need to supply data that conform to such standards will increase the dependence of experimental laboratories on data repositories. Such repositories may be accessed within the laboratory as well as by collaborators or the wider research community.

Developing effective interfaces to bioinformatics resources requires consideration of the limitations imposed by the working environments in which these interfaces are created. Laboratories may second personnel to maintain data repositories. These repositories often begin as prototype services [1]. Laboratory personnel are trained in their domain science but are rarely trained software engineers. Moreover, repositories may be designed for specific, short-term goals, but then may come to be depended upon by a larger community that has different goals, necessitating software evolution. Such development activities are often of an *ad hoc *nature and yield interfaces that are not robust, and are difficult to evolve or maintain once the associated personnel have left.

The focus of this paper is the development of a systematic, model-driven approach to the construction of user interfaces to bioinformatics repositories. With this approach, many aspects of software infrastructure are specified using declarative models, and executable programs are generated from such models. This approach adapts a design paradigm called the Model Driven Architecture (MDA) [2], which has had less exposure in bioinformatics than in other domains.

The remainder of this paper describes the model-driven approach to software development. We indicate how this has been adopted in the development of a system called Pierre, which uses models to construct and generate user interfaces to bioinformatics resources.

### Model Driven Software development

#### Spectrum of approaches

Software development methods can be characterised by the relationship between a model and an application's code base [3]. These methods are ordered, below, by the increasing role of models in descriptions of software behaviour.

• *code-only*: no models describing the system are developed. In this approach, the code is the only artefact that results from a development cycle. Any design abstractions used are expressed solely in the structure of code components.

• *code-visualisation*: uses software tools that associate graphical modeling notations with views of code bases. Such tools allow programmers to manipulate notations rather than code. For example, button elements could be altered in size graphically, which has the effect of setting dimension properties in the code.

• *round-trip engineering (RTE)*: uses an abstract model of the system to help guide development of the code base. The model is manually implemented during the design phase prior to the implementation. Designs that are captured in models can be enhanced by refinements in implementation, and models should reflect such refinements. In this approach, models must be synchronised with their associated code bases, and the model serves as part of the system-level documentation.

• *model-centric*: the models are developed in sufficient detail to allow them to be used to derive the code base. Developers refine the models through a series of transformation steps that ultimately yield program code.

• *model-only*: models are developed in sufficient detail to allow code bases to be derived from them. Models are refined through a series of transformation steps, resulting in program code. This approach is employed when a project's goal is to develop models for later use in creating implementations.

User interfaces to bioinformatics resources are developed in environments that typically do not easily facilitate these approaches. Prototyping can identify short-term requirements for development, and the code written for these is often not well-structured. Experimental environments that evolve rapidly emphasise the exploration of new techniques and not the support of software products. Within such environments, prototyping is an activity that works well. However, prototypes are often used as the end product, rather than as a stage towards an end product. In this event, requests for capability enhancement can result in code bases that are difficult to maintain.

Code visualisation is a technique that provides a graphical framework within which a code base is developed. However, this technique may be too limited to capture important features of the design. For example, some visualisation tools focus on allowing developers to build user interfaces using predefined classes for features such as buttons, scroll bars, and text fields. Developers provide the detailed functionality of the applications by filling in stubbed call-back methods associated with these features. In such an approach, however, rather little of the functionality of the application may be represented in the visualisation.

RTE assumes developers have enough skill, time, and discipline both to create models and to synchronise them with code bases. This approach may also require developers to create an analysis model, a design model, and a code base. RTE is unlikely to be sufficiently responsive for use in rapidly changing contexts, such as bioinformatics laboratory environments.

The model-only approach is used by standards bodies in bioinformatics for the purpose of producing models that describe data sets for a particular biological domain. Their aim is to produce models that describe the data sets for some particular biological domain [4,5]. With this approach, developers are delegated to creating and maintaining data repositories and interfaces, both of which are expected to be compliant with a model. However, this approach leads to software applications that evolve independently of community models.

The model-centric approach has a number of characteristics that are well suited to environments in which bioinformatics tools are produced. Model-centric applications are designed to accommodate change: model changes, which are reflected in code changes. This process also tends to be done automatically, which helps to reduce the number of errors in code bases. Distinctions can be made between modeling and developing, but with this approach, modelers may find themselves engaging in development activities. This allows laboratory personnel to focus on making contributions based on domain knowledge rather than attempting to perform software engineering. When additional programming is required, it can focus on customised features rather than on generic ones. The model-centric form of development supports rapid prototyping activities that allow end-users to validate models. This validation is done by means of feedback on automatically generated applications. The result of using this approach is that time spent prototyping is reduced and time spent adapting applications for production environments in increased.

#### Model Driven Architectures

The model-centric approach is embodied by the Object Management Group's Model-Driven Architecture (MDA) [2]. The main aim of MDAs is to support software development through the application of transformations to various kinds of models. These models are listed below in order of their decreasing level of abstraction.

• *computation-independent model *(CIM): represents concepts from domain experts' perspectives. For example, concepts such as Protein, Modification, or Gel may be used by the CIM to describe proteomics schemata.

• *platform-independent model *(PIM): describes system aspects that are independent of deployment activities. PIMs describe tasks supported by software applications but do not include implementation details. Examples of PIM concepts include *Editing*, *Searching *and *Browsing*

• *platform-specific model *(PSM): describes implementation-specific details of given deployment environments. An example PSM could include details of how specific features are supported in web applications.

The term *platform *can have various meanings, and can include one or more system aspects such as operating system, network configuration or programming language. More important than the definition of *platform *is the notion that the PIM addresses application logic and separates this from the PSM, which addresses implementation details. A central idea of MDA is that transformations can be applied to convert a CIM to a PIM and finally to a PSM. The PSM is supposed to contain sufficient information about domain concepts, software features and platform details to facilitate the automatic generation of software code [3].

Model-Based User Interface Development Environments (MBUIDEs) [6] have been developed for user interface development using techniques that are closely related to OMG's MDA but that either do not follow OMG explicitly or predate OMG. For example, the MIDAS project [7] proposed a model-driven methodology for the development of Web Information Systems (WISs), and Teallach [8] supported model-based user interface development for databases. However, MBUIDEs have not been widely adopted in practice, probably for a combination of reasons. These reasons may include the following factors: (1) the collection of models used to develop comprehensive interfaces is complex; (2) the emphasis of many proposals is task-model centred, suiting only certain styles of interface; and (3) models for describing user interface functionality have often been poorly integrated with other models used as part of the software development process. These issues are addressed in this paper by adopting model-driven techniques in a narrowly defined domain. In this way, models are straightforward, tasks are drawn from a fixed set, and data repositories are accessed through consistent interfaces.

A few projects have applied this paradigm specifically to bioinformatics. For example, MEMOPS [9] presents a framework for scientific data modeling and automated software development. This framework was originally developed to suit applications for NMR spectroscopy, although its authors claim it is general enough to apply to other domains. The focus of MEMOPS appears to be the application of model-centric approaches to automatically generating software that can read and write data expressed in various formats. The framework promotes the idea of client programs interacting with an application program interface (API) rather than directly with a particular data format.

This paper's focus is the use of the model-driven approach, as supported by Pierre, for automatically generating interfaces for accessing data repositories. This follows on from earlier work by the authors on the Pedro system [10]. Pedro generates data capture forms directly from an XML Schema, which describes the structure of data to be captured, and from additional configuration information provided by Data Modelers. Examples of configuration information are context-specific help and controlled vocabularies, both of which can be used to guide data capture. However, Pierre adopts a model-centric approach more comprehensively than does Pedro. Pierre supports an interactive design phase through which PIMs are developed. These PIMs may, in turn, be associated with PSMs for both creating different styles of interface and accessing different kinds of repository.

## Methods

This section describes the scope, design, implementation and use of Pierre. Success in applying MDAs has so far depended on the following:

• use of declarative models for the identification of use-case patterns that lend themselves to description;

• development of models that directly capture common behaviours;

• design of effective interfaces between models and existing components;

• and provision of effective tools for model construction and application generation.

In Pierre, we address these aspects by:

• Identifying four common access patterns to data repositories: browsing; simple searching using canned queries; advanced searching using user-defined filtering based on predicates; and expert searching based on direct use of query languages. Individual interfaces can support any combination of these categories of data access.

• Developing models specific to each data access category, whereby application-specific interfaces are described using a manageable number of modeling decisions.

• Designing an open architecture that allows Pierre applications to interface to existing data management systems, security models and ontology services.

• Providing a service configuration tool that supports both immediate review of interfaces and generation of applications implementing different interfaces. The interfaces are supported by the underlying model.

**Figure 1:**
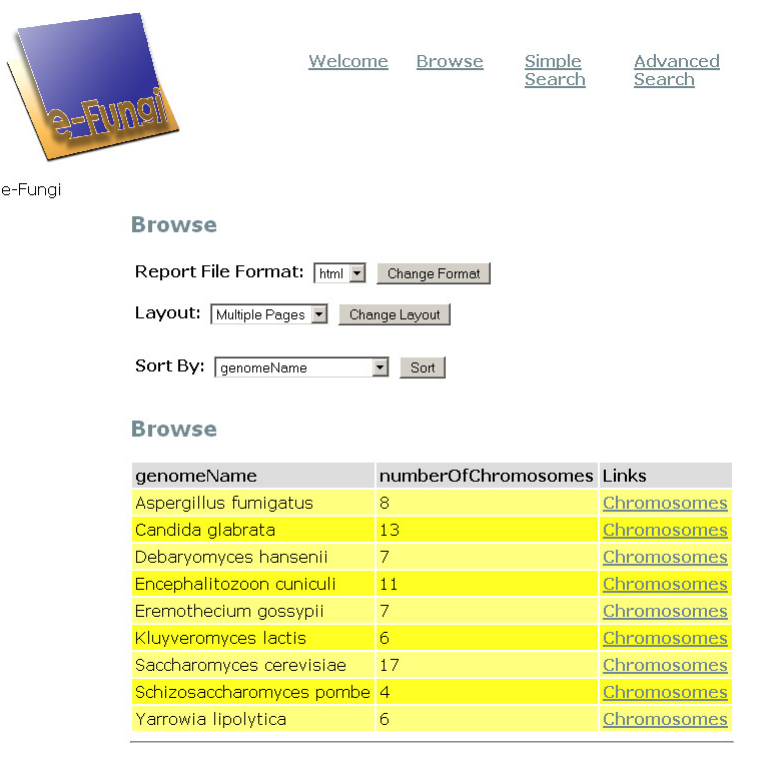
The e-Fungi *Browse *interface using the web as the deployment platform. As e-Fungi supports analyses over multiple fungal genomes, the display lists the genomes represented in the database, with the option to obtain additional information on their chromosomes.

**Figure 2:**
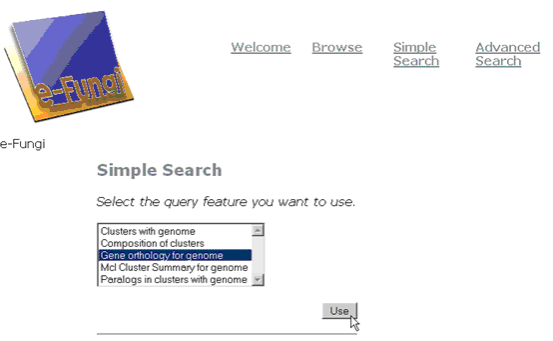
The top-level e-Fungi simple search interface using the web as the deployment platform – a list is provided of the available queries.

**Figure 3:**
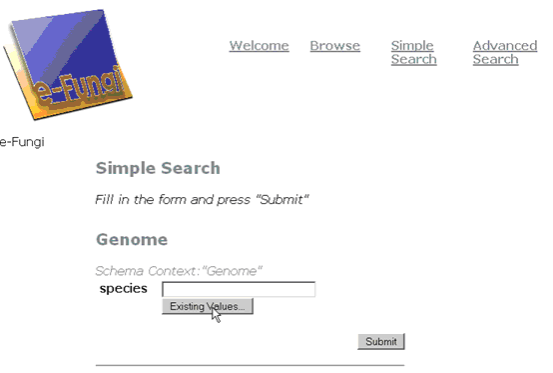
A specific simple search page that prompts the user for a parameter for a specific search; the permitted values for the entry can be retrieved from the database.

**Figure 4:**
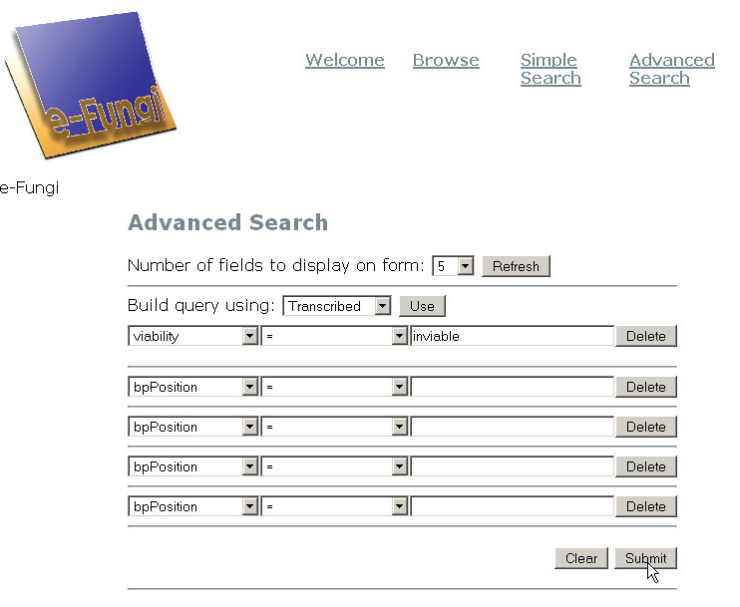
The e-Fungi advanced search interface using the web as the deployment platform to search for essential genes.

**Figure 5:**
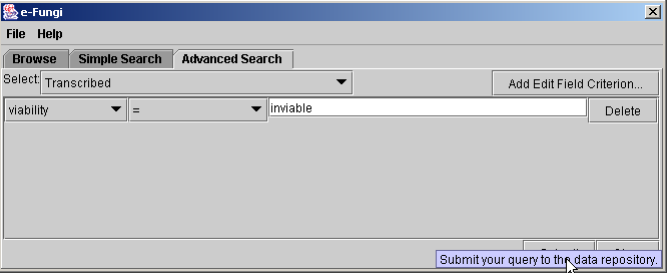
The e-Fungi advanced search interface for the desktop application, showing the same request as for Figure 3.

An example of a Pierre deployment is the user interface to the e-Fungi database, which contains sequence and functional data from multiple fungal species. From the web version of the e-Fungi deployment, Figure 1 illustrates the Browse interface, which lists properties of the genomes in the database, and allows users to navigate to obtain further details. Figures 2 and 3 illustrate the Simple Search interface. Figure 2 shows how a list of canned queries is made available, each of which is associated with a form that collects the parameters for a search, as illustrated in Figure 3. Figure 4 illustrates the Advanced Search interface, in which a search is configured. This is done by identifying a collection over which the search is to take place and the predicates that are to be used to filter the objects in the collection. Figure 5 also shows the Advanced Search interface, but this time for the stand-alone application.

## Results

### Pierre as a Model-Driven Architecture

**Figure 6:**
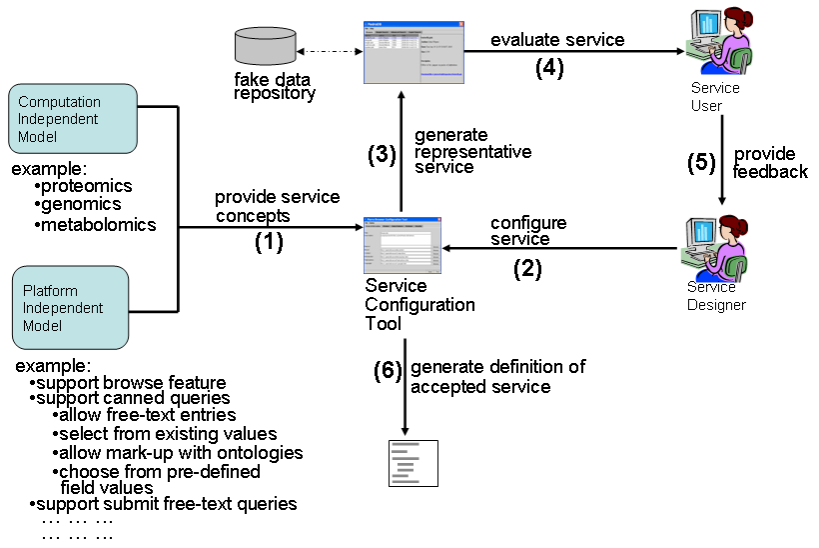
The rapid prototyping phase of Pierre. The principal components in the Pierre model-driven architecture. Round cornered boxes represent models, rectangles represent software components, solid lines represent interactions between software components and dashed lines represent human interactions with software.

The use of the Pierre system involves two phases: a rapid prototyping phase and a deployment phase. Figure 6 shows how a service designer builds a service using the *Service Configuration Tool; *the numbers in brackets are explained below. The tool is driven by concepts provided by a *Computation-Independent Model *(CIM) which is expressed as an XML Schema [11] and a *Platform Independent Model *(PIM) which is expressed in XML. The CIM contains domain concepts such as "protein", "gene", "sample" and "experiment" that are relevant to the application. The CIM typically describes the schema of the database to which an interface is being provided. These concepts can be associated with various form generation properties described in the PIM. For example, the PIM could describe whether a form field supported free-text entries as well as selection based on existing values. It could also describe properties of features such as canned queries, queries that could be dynamically constructed by users and a facility for submitting free-text queries.

These two models provide concepts that drive the operation of the tool (1). A *Service Designer *uses the *Service Configuration Tool *to iteratively build a description of how the dissemination service should behave (2). That is, the PIM is created using the Service Configuration Tool. The designer can then auto-generate a representative service to show service users (3). This service communicates with a fake data repository that returns random data. The service is intended to provide enough information for service users to evaluate the prototype (4) without requiring that a finished data repository has already been developed. The users convey their feedback back to the service designer (5), who then modifies the definition of the service. When the *Service Users *accept the prototype, the service designer can use the configuration tool to generate a definition of the service.

**Figure 7:**
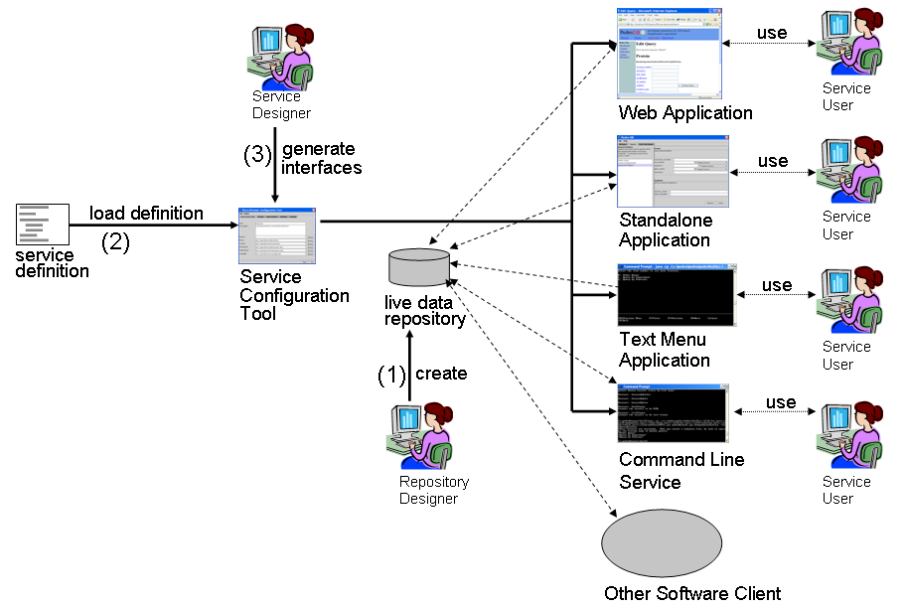
The deployment phase of Pierre. Arrows indicate steps used to auto-generate query interfaces. Dashed lines indicate communication between deployments and the data repository or communication between the deployments and the users.

The deployment phase is shown in Figure 7. During or after the service is prototyped, the *Repository Designer *creates a live data repository (1). Pierre has been used with XML databases containing cell image metadata and proteome experimental results, a relational database containing medical data, and the e-Fungi object database. The *Service Designer *can use the *Service Configuration Tool *to auto-generate a human readable description of what the service can do. This functional specification can aid the *Repository Designers *in their task of creating a repository that responds to the needs of the service users. When the back-end of the service is completed, the *Service Designers *can configure the service to substitute the fake repository for the live one. They can then use the *Service Configuration Tool *to automatically generate multiple deployment forms (3) based on the same service definition (2).

In MDA terminology, each deployment is regarded as a *Platform-Specific Model *(PSM). The approach advocates expressing a PSM as a configurable model that can be used to auto-generate code. However, in Pierre, properties of the PSMs are fixed and reflected in code rather than in formal models. This is done to make the *Service Configuration Tool *simpler to use by the *Service Designers*.

**Figure 8:**
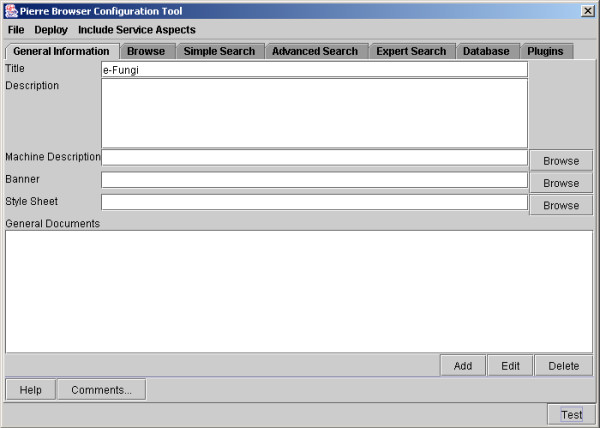
Top-level interface to the Pierre Model Editor, showing tabs for each of the principal kinds of user interface capability.

**Figure 9:**
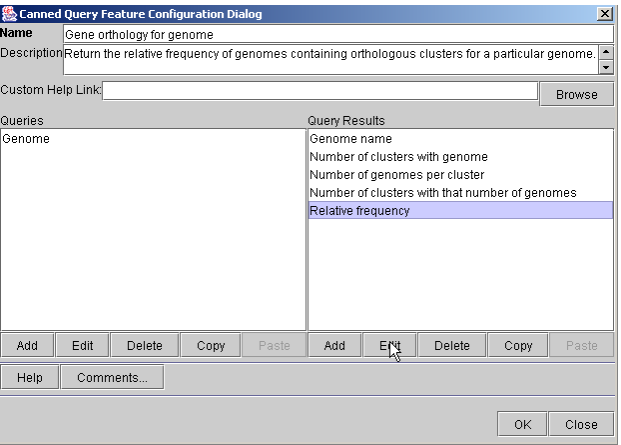
Editing a Simple Search form for the query in Figure 3 using the Pierre Model Editor. The input and output of the query are specified separately. Here, a field called "Relative frequency" is added to the output to display frequency values that are calculated as result of the query.

**Figure 10:**
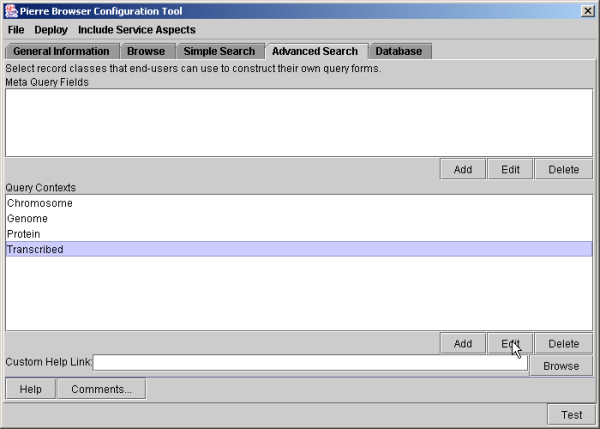
Editing the Advanced Search interface for e-Fungi using the Pierre Model Editor. Classes that users are allowed to query are added. The resulting interface is shown in Figures 4 and 5.

The Service Configuration Tool supports the tasks described above. Its top-level interface is illustrated in Figure 8, which indicates the different capabilities that the Service Developer can configure. The use of the Service Configuration Tool to design a Simple Search is illustrated in Figure 9, and the design of an Advanced Search is illustrated in Figure 10.

Users will eventually endorse a prototype service, which will become the initial production version, allowing the Service Designer the option to enter the deployment phase of development. The Service Configuration Tool uses a description of the service to automatically generate an Ant [12] script. This script is then run and multiple deployment forms are automatically generated: web-based, stand-alone, text menu and command line. Each deployment interacts with the live data repository through an API. This API can also be used by other software clients that wish to use data provided by the service. To support the integration of deployments in complex sequences of tasks, the stand-alone application can also be invoked as a component by other software clients. The web deployment that is generated is a folder that is meant to be placed in the web applications directory of a Tomcat instance.

### Pierre as an Open Architecture

**Figure 11:**
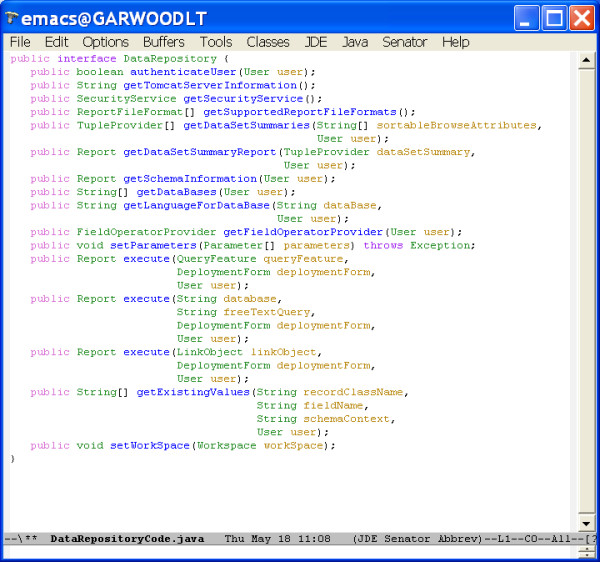
The *Data Repository *interface that is implemented or extended by *Service Developers *to provide access to different kinds of data repository, or to support analyses over a specific repository. For example, a front-end application can pass a free-text SQL query to the execute method having the parameter "freeTextQuery". The implementation will then apply this query to an underlying database and return the results in a report.

In the MDA approach, generic program features are supported through model-driven activities and specialised program features are supported by a collection of services known as a Service Oriented Architecture (SOA). Developers customise an application by implementing one or more service interfaces as software plugins. The types of services that are supported represent aspects of the domain use case that warrant customisation. These areas were identified for data dissemination applications used in bioinformatics:

• *reports*: display results of querying and browsing; default representations are provided. Service Developers can augment standard representations for such things as new layouts or specialist visualisations for specific kinds of data, such as multiple sequence alignments.

• '*links*: cross-references from Pierre reports to other sources of information. A link may lead to an external information source or may implicitly cause more refined queries to be applied to the repository. Service Developers can augment standard types of links with navigation implementations. For example, the ability to navigate to a web page may be adapted so that links evaluate follow-on queries over the data repository.

• *ontology services*: ontologies or controlled vocabularies associated with specific search fields in Simple and Advanced Search. Ontologies may be maintained using different technologies, and Service Developers can augment the ontology services provided by Pierre to obtain terms from custom ontology servers or formats. Pierre's Ontology Services are managed using the Pedro Ontology Services Framework [13].

• *security*: mechanisms used by applications to control the release of information. Service Developers can include custom mechanisms for authenticating users and for authorising specific tasks, such as the evaluating specific queries.

• *validation*: supplied by most Pierre capabilities as simple form field type-checking, as in Simple Search and Advanced Search. Service Developers can augment the validation provided to support custom checks, such as verifying that keys comply with regular expressions or do not appear in external databases.

• *data repository*: the most important point of extensibility. All repositories are accessed by implementations of an abstract repository interface illustrated in Figure 11. Repository Designers have developed deployments that access relational databases (in particular, mySQL), XML repositories (in particular, eXist) and object databases (in particular, FastObjects) through extensions to the data repository interface. Furthermore, a single data repository service could access multiple data repositories, for example through some form of distributed querying infrastructure, although Pierre itself does not directly support data integration.

**Figure 12:**
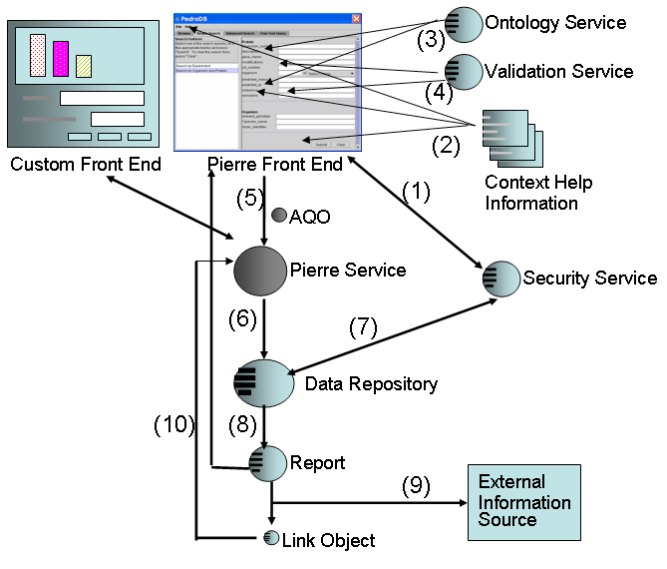
Roles of the extensible components in the architecture of Pierre. Arrows indicate steps for submitting a query. Context Help Information and External Information Source represent documents that can be referenced by the query service. Spheres with slots indicate interfaces that can be implemented by developers. The Pierre Service and AQO objects are part of the core architecture and are not configurable by developers.

These aspects of extensibility are supported as service classes within Pierre. Figure 12 shows how these services augment the behaviour of a deployment during the course of a query submission activity. Initially, a query form is auto-generated by the *Pierre Deployment*. It may consult a *Security Service *to determine what form features are appropriate to display for a given *Service User *(1). Before specifying a query, the users may want to access *Context Help Information *(2) which could describe the meaning of form concepts. As they fill in the form, they may want to mark up some fields with terms provided by one or more *Ontology Services *(3). When they submit the query, *Validation Services *(4) are invoked to check for any errors.

When the query is correctly specified, the deployment bundles the form values into an Abstract Query Object (*AQO*) (5). The *AQO *holds a canned query and contains data structures that correspond to concepts defined in the PIM. The *AQO *is received by a *Pierre Service*, which uses a published API to interact with auto-generated deployments as well as other custom deployments. The *Pierre Service *forwards the *AQO *onto the *Data Repository *(6), which executes the query and retrieves results from a database management system. The *Data Repository *may consult the *Security Service *(7) to determine what kinds of results are appropriate to show a given *Service User*. The repository assembles results into a *Report *(8) that is returned and displayed to the user.

**Figure 13:**
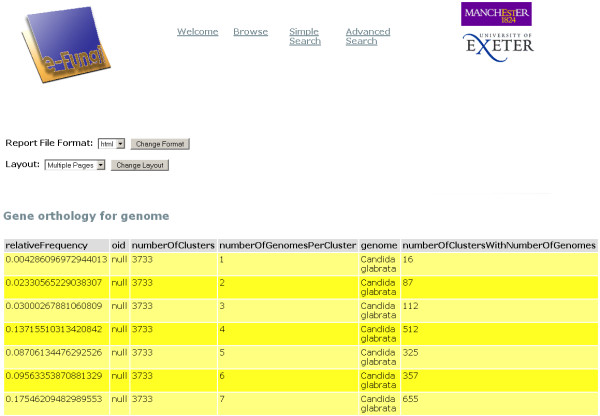
The result of the query from Figure 3.

The *Service User *may access a link displayed in the *Report *to request further information. Figure 13 shows how an implementation of a Report service renders the results of a search in tabular form. The link mechanism is represented by a *Link Object*, which may have different representations for different deployments. For example, it may present a hyperlink in the standalone or web deployment but may present a menu number in the text menu application. The *Pierre Deployment *will ask the *Report *if it knows about a *Link Object*. If the link is not known to the *Report*, it is assumed to be a link to an *External Information Source *(9). For example, in the web deployment, a hyperlink could refer to another web site. If the link is known to the *Report*, the *Pierre Deployment *assumes the object represents a request to obtain more information from the *Data Repository *(10). The *Link Object *is forwarded to the *Data Repository *via the *Pierre Service*. The *Data Repository *uses parameters defined in the link to construct a follow-on query, and then produces a *Report *in the same manner as before.

The AQO and Pierre Service are used to foster communication between the Pierre Deployments and the Data Repository. The remaining objects are all services that can be customised by software developers. Pierre comes with default implementations for all services, although the implementation of the Data Repository typically requires some coding effort to tailor it for a specific application. In particular, the construction of a suitable top-level representation of the contents of a repository for the Browse interface normally requires some manual coding, and the queries made available through the Simple Search interface have to be implemented by the developer. By contrast, the default implementations of Advanced Search and Expert Search can be expected to work with little configuration. For example Pierre deployments supply Data Repository components for the Advanced Query interface that support translation from interactive representations to query languages of the underlying database. Implementations of such translations have been developed for SQL, JDOQL [14] and XPath [15].

### Deployments

Pierre has been used to create interfaces for three kinds of data repositories. Pierre documentation for the Service Designer uses a tutorial model based on cancer patient medical records, the data for which are stored in a MySQL database. Documentation for Pierre addresses several roles identified for the creation of a repository interface, and this documentation is available online or with the Pierre download. The second use case was to rework the interface to PEDRoDB [16], which is a database of experimental proteomics data captured using the model from [17]. This model is represented in an XML Schema, and has been implemented using the eXist [18] native XML Database System. The third use case was to develop the web interface to the e-Fungi data warehouse. This data warehouse is used to support comparative analyses of fungal species and is an evolution of the GIMS [19] database. The e-Fungi use case has been implemented with an object database using the JDO application programming interface to FastObjects.

The results of applying the tool are encouraging. The successful deployment of the tutorial database shows that a system using an XML representation of the database can be applied to work with relational databases. The auto-generated web application used for PedroDB supports greater functionality than that described in [16], and shows that Pierre can be applied effectively over existing databases. Here, also, Pierre is used directly with native XML databases, which may become widely used for storing standardised data. The e-Fungi interface confirms the utility of this approach for a diversity of both domains and data management technologies.

## Discussion

The objective of the Pierre project is to use model-driven development techniques to achieve several defined goals. We want to support functionality requirements from multiple domains. We also want to reduce the amount of time spent on common interface programming tasks in the hope that the time saved allows resources to be better deployed to the specifics of projects. Moreover, this project facilitates the development of applications that are robust enough to support the demands of a production environment. This section reviews Pierre in the context of these criteria and discusses related work.

### Functionality

Pierre produces data access interfaces that have functionalities comparable to those interfaces supported by many public bioinformatics databases (e.g. [20,21]). This is not surprising, as the features supported for model-driven generation in Pierre are those identified by the authors as representing important recurring themes. Pierre supports the inclusion of interface functionalities that are necessary, but not always sufficient, in many contexts. In principle, Pierre could be used outside bioinformatics, but the requirements supported have been gleaned from studying user interfaces in bioinformatics, and we anticipate that there will be significant functionality gaps if Pierre is deployed in unrelated application areas.

Generic interfaces that support common behaviours cannot compete with interfaces produced by well-resourced specialised development activities, such as those associated with genome sequencing activities [22]. However, we contend that there are serious limitations to the development resources that many bioinformatics researchers are able to commit to the construction of interfaces to potentially important data resources; thus that Pierre addresses a common need. In addition, if a site develops multiple interfaces using Pierre, this will increase consistency between the interfaces, thereby reducing complexity and learning times for users.

### Developer resources

The system design methodology supported by Pierre can save time in at least five ways. First, it reduces the time programmers spend manually coding prototypes. Second, parallel development of the front and back ends of a query service is facilitated by the decoupling of front-end concerns from back-end concerns. Third, the requirement to develop interfaces for common cases is removed. In practice, creating a data access application involves straightforward, but time-consuming, interface programming, often in environments with steep learning curves. Fourth, the fact that Pierre can generate deployments that use different delivery platforms removes the need to develop and maintain code bases for multiple platforms. Fifth, as less code is developed for a specific deployment, the times both testing and fixing such code is reduced.

It is difficult to make general statements about how long it takes to develop a deployment using Pierre. Most of our current deployments were produced at the same time as the associated repository services, significant components from which can be reused in future deployments. However, we note that Pierre supports incremental development, and that the time taken to develop a preliminary deployment can be modest. For example, to create a deployment over a relational database, the following steps are required:

1. Create a CIM that represents some or all or part of the schema of the database; a tool is provided for this purpose.

2. Load the resulting CIM into the Service Configuration Tool, and generate a default deployment.

The default deployment will include only Advanced Search and Expert Search, but should be able to be created in a small number of hours. This assumes that the database already exists, the relevant software is installed, and the developer is familiar with Pierre. Thereafter, the creation of a Browse interface involves a small amount of design with the Service Configuration Tool, and the implementation in Java of an interface provided with the Repository Service. Depending on the complexity of the Browsing to be supported, this task should take from a few hours to a few days. The development of a query for Simple Search involves a small amount of design with the Service Configuration Tool, and the implementation in Java of the associated search in the context of the Repository Service. Depending on the complexity of the Search to be supported, this task should take from a few hours to a few days. As such, having learned how to use the Pierre system (which might be expected to take a week), a deployment over an existing relational database, including a top-level Browse interface, 10 queries in the Simple Search, Advanced Search and Expert Search should be able to be developed in a small number of weeks. Of course, a typical development activity involves significant effort on requirements capture, including iterative design steps. However, the work required on the implementation of a service comparable to the e-Fungi interface illustrated in Figures 1 to 6 should involve weeks rather than months of effort.

### Robustness

Pierre generates most of a deployment's executable code. This allows for systematic testing of the code base to be conducted, and revisions, which are suggested by multiple deployment communities, to be incorporated in a structured way. Furthermore, developer effort associated with a specific deployment is focused on certain tasks. This enables developers to take a systematic approach to testing of both custom analysis features and repository capabilities.

### Related work

The application of techniques from model-driven architectures has allowed Pierre to improve the efficiency of interface development for bioinformatics resources. This section focuses on comparing and contrasting Pierre with other generic infrastructures used for developing bioinformatics interfaces. Generic infrastructures are defined here as those infrastructures that are not associated principally with a single data repository or a single domain within bioinformatics; we focus in particular on BioMart [23] and SRS [24].

Like Pierre, BioMart supports development of customised interfaces to bioinformatics databases, including the construction of advanced search interfaces. The principal difference in ethos between Pierre and BioMart is that the latter is designed for use over relational databases implementing a variation of star schema models from data warehouses. As such, BioMart encourages and exploits a specific way of representing the data over which interfaces are to be built, and thus may be less suitable than Pierre for use with existing databases. Given a suitable schema, BioMart provides many configuration options, which cover aspects of security, report linking and output file formats. Pierre provides fewer configuration options than BioMart, but retains considerable flexibility through an open architecture with many extensibility points. While BioMart provides a flexible infrastructure for development, it integrates the design of the repository and the repository interface more closely than does Pierre. As such, we assess Pierre as allowing repository designers greater flexibility in their choices by separating interface design and development from data management.

SRS is a well established infrastructure, principally designed to support the development of navigational interfaces to flat-file repositories, although other data repositories can also be accessed using this infrastructure. However, SRS continues to have a principal focus on linked collections of file-based resources, which are indexed to support efficient access and navigation. As a result, SRS provides both management and integration capabilities, and thus focuses more on enterprise-level information management than does Pierre. In contrast, Pierre's principal use is to construct interfaces to individual data resources, often where these resources exist already and where the effort in creating or maintaining bespoke interfaces is felt to be problematic.

## Conclusion

This paper has described an architecture that increases the efficiency of development of interfaces to bioinformatics data resources. This is done by identifying recurring requirements that such interfaces must support, and by adopting a model-driven architecture to support these requirements. Developers use a Service Configuration Tool as a means of manipulating the underlying model in order to specify interface characteristics for specific kinds of data. Development efficiency is also increased by supporting an open architecture, whereby components may be replaced or extended to augment default behaviours.

The overall approach has been implemented in a system known as Pierre. This, in turn, has been used to construct user interfaces to several bioinformatics data repositories implemented using different kinds of database management systems. The Pierre system, with its model-driven approach, greatly reduces the amount of time spent on re-addressing the routine aspects of interface development. This enables domain experts to make meaningful contributions to the development process based on their true expertise.

## Availability and requirements

The Pierre Project is described at: . The Pierre code base is maintained on Source Forge and is distributed under the Academic Free License. The software has been tested on Windows-based environments and currently depends on JDK1.4 and Ant 6.0. The tutorial repository requires that MySQL is installed. Automatically generated web applications work with an environment that uses Tomcat 5.0 and Apache 2.0. The three example databases referred to in this paper may be linked to from .

## Authors' contributions

KG is the main software architect for the Pierre Project and initially drafted the paper. CG has been responsible for creating the repository data model for the tutorial and adapting the PedroDB query service to suit Pierre. He is also responsible for end-user training, and development of test plans for the project. He, together with SGO, proof-read the draft developed by KG and NP. CH used Pierre to develop the e-Fungi deployment. TG provided feedback on an early version of Pierre. NS developed the generic Data Repository service for relational databases. SGO oversees the life science aspects of the projects that motivated the development of Pierre and thus provided a user's perspective. NP managed the project, helped to steer development of the architecture, and ensured the paper targeted bioinformaticians. All authors have read and approved the final manuscript.
